# Origin of deformed halite hopper crystals, pseudomorphic anhydrite cubes and polyhalite in Alpine evaporites (Austria, Germany)

**DOI:** 10.1007/s00531-012-0836-6

**Published:** 2012-12-07

**Authors:** C. Leitner, F. Neubauer, R. Marschallinger, J. Genser, M. Bernroider

**Affiliations:** 1 Fachbereich für Geographie und Geologie, Universität Salzburg, Hellbrunnerstraße 34, 5020 Salzburg, Austria; 2 Institut für Geographic Information Science, Österreichische Akademie der Wissenschaften, Schillerstrasse 30, 5020 Salzburg, Austria

**Keywords:** Evaporites, Halite, Polyhalite, Ar–Ar dating, Alps

## Abstract

The Alpine Haselgebirge Formation represents an Upper Permian to Lower Triassic evaporitic rift succession of the Northern Calcareous Alps (Eastern Alps). Although the rocksalt body deposits are highly tectonised, consisting mainly of protocataclasites and mylonites of halite and mudrock, the early diagenetic history can be established from non-tectonised mudrock bodies: Cm-sized euhedral halite hopper crystals formed as displacive cubes within mud just during shallow burial. The crystals were deformed by subsequent compaction. Later, migrating fluids led to the replacement of halite by anhydrite retaining the shapes of deformed halite cubes. Polyhalite formed from subsequent enhanced fluid migration. Mudrock provided water by dewatering, while potassium and magnesium were dissolved from primary salt minerals. When these fluids interacted with sulphates, polyhalite precipitated. ^40^Ar/^39^Ar analyses date the polyhalite from within the retaining shapes of deformed halite hopper-shaped cubes from two localities to ca. 235–232 Ma (Middle Triassic). At this time, ca. 20–25 Ma after sedimentation, polyhalite crystallised at shallow levels.

**Electronic supplementary material:**

The online version of this article (doi:10.1007/s00531-012-0836-6) contains supplementary material, which is available to authorized users.

## Introduction

In Alpine Haselgebirge evaporites, halite hopper crystals occur, which have been replaced by anhydrite and/or polyhalite. The reconstruction of the replacement stages gives insight into their early diagenetic evolution. Although the Haselgebirge salt contains ca. 50 % mudrock and stands alone within the international literature, such type of salt rocks may well be expected in other regions of the world, but may have not been payed much attention due to the lack of economic interest. The conspicuous deformation of halite hopper crystals, pseudomorphs of anhydrite after halite and polyhalite crystallisation must be seen strongly in the context of the large proportions of mudrock and anhydrite-mudrock, respectively. This study contributes to a general understanding of diagenetic processes within mudrock-dominated salt.

Halite hopper crystals and imprints of halite cubes in fine-grained clastic sediments are a common feature of evaporitic sequences (e.g. Görgey 1912; Linck 1946; Haude 1970; Gornitz and Schreiber 1981; Casas and Lowenstein 1989; Benison and Goldstein 2000; Kendall 2000; Pope and Grotzinger 2003). Halite hopper crystals in mudrock are characteristic for all Alpine rocksalt deposits including Altaussee, Berchtesgaden, Bad Dürrnberg, Hall in Tirol, Hallstatt and Bad Ischl (Schauberger 1931; Westner 1951; Schindl-Neumaier 1984; Schauberger 1986; Spötl 1988a). This rock type is traditionally referred to as “Tonwürfelsalz” (also: “Kropfsalz”) in the Alpine salt mining literature. Halite cubes from Alpine localities were first described by Haidinger (1847). In his interpretation, the cubes grew in soft mud and suffered subsequent deformation during compaction (1847, 1853). Görgey (1912) found similar rocks in Wittelsheim, Ober-Elsaß (France). This Oligocene rocksalt deposit suffered no tectonic deformation and the maximum overburden was 500–1,000 m (Hinsken et al. 2007). Still, cubes in these rocks show the same distorted shapes as those in the highly deformed Alpine deposits. Görgey postulated a primary growth by fluid migration without later deformation.

In Alpine rocksalt deposits, cubes of anhydrite, and partly of polyhalite, also exist within mudrock. The anhydrite cubes of Berchtesgaden are unique within the Alpine rocksalt deposits with respect to their size and number (mentioned by Westner 1951; Kellerbauer 1996). Anhydrite and halite cubes expose the same features including spatial arrangement within the mudrock and shape geometry. At Bad Dürrnberg and Hallstatt, similar cubes also occur, and we found anhydrite veins of the same rose colour and halite cubes marginally substituted by anhydrite. Haidinger (1840, 1847) reported anhydrite cubes from Bad Dürrnberg and Hall in Tirol. In the international literature, anhydrite cubes are only rarely described and we found only a single description of pseudomorphs of calcite after anhydrite (Kowalczyk 1975). Pseudomorphs after halite cubes, replaced by dolomite (Pope and Grotzinger 2003), and by marcasite, quartz and calcite were also reported (Martill et al. 2007).

A further characteristic of the mudrock-dominated Alpine rocksalt deposits is polyhalite. The composition of polyhalite, K_2_Ca_2_Mg (SO_4_)_4_·2H_2_O, was first analysed in samples from Bad Ischl (Stromeyer 1818), and the crystal structure was first determined in samples from Altaussee (Schlatti et al. 1970; later confirmed by Bindi 2005). Under laboratory conditions, polyhalite can be synthesized by the reaction of gypsum with appropriate solutions at temperatures above 70 °C (Freyer and Voigt 2003). In the wet hexary system Na^+^, K^+^, Mg^2+^, Ca^2+^/Cl^−^, SO_4_^2−^//H_2_O, polyhalite is also stable at room temperature (Wollmann 2010). Under laboratory conditions, the dehydration process of polyhalite starts at 255 °C and is completed at 343 °C (Wollmann et al. 2008). As will be argued in the present study, polyhalite precipitated from migrating fluids through the mudrock and mudrock-anhydrite sequences. It thereby also partly replaced halite within deformed halite hopper crystals.

The localities of these halite-anhydrite-polyhalite cubes are part of the Haselgebirge Formation and expose peculiar features: (1) extreme tectonic deformation, (2) a large proportion of mudrock and (3) the absence of K-bearing evaporite minerals other than polyhalite. They were subjected to temperatures of ca. 180 °C in Hallstatt (Spötl and Hasenhüttl 1998) and more than ≥250 °C in Bad Ischl, Altaussee (Wiesheu and Grundmann 1994) and Berchtesgaden (Kralik et al. 1987). Similar temperatures were found in gypsum/anhydrite deposits of this unit. The most reliable temperature measurements are based on fluid inclusions in quartz, which revealed 220–260 °C (Spötl et al. 1998).

An important aim of this study is to explore the origin of deformed halite hopper crystals. However, the major focus is to constrain the conditions under which anhydrite replaced halite within the “cubes” and the timely more or less equal crystallisation of polyhalite. Field work in the salt mines of Altaussee (ALT), Berchtesgaden (BDG) and Bad Dürrnberg (DÜ) was combined with X-ray computed tomography, electron microprobe analysis and ^40^Ar^/39^Ar age dating.

## Geological setting

**Fig. 1:**
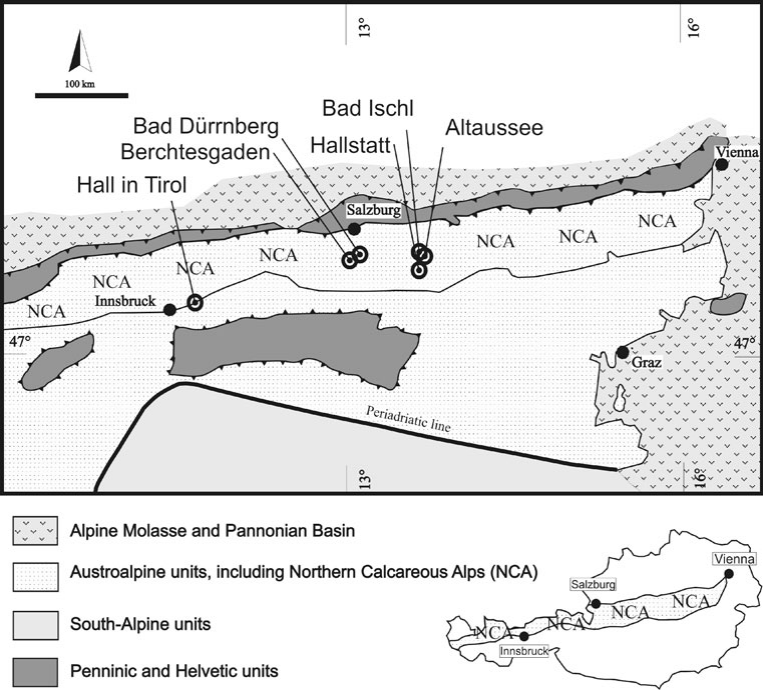
Overview of the Eastern Alps. The main tectonic units and the location of the NCA. *Circles mark* Alpine rocksalt deposits

The Northern Calcareous Alps (NCA, Fig. 1) form a fold-and-thrust belt within the Austroalpine unit of the Austrian and German Eastern Alps. The classic division within the Northern Calcareous Alps defines the Bajuvaric, Tirolic and Juvavic nappe complexes. The age of formations in the NCA ranges from the late Carboniferous (?) or early Permian to the Eocene. Rocksalt deposits are mostly found in the Lower Juvavic unit. At the transition from the Lower to the Upper Cretaceous nappe stacking of the Austroalpine units began as a result of the subduction of the Austroalpine continental crust. The classic model assumes that both Juvavic nappes were emplaced during eo-Alpine nappe tectonics (Mandl 2000). Thrusting propagated from southeast to northwest, respectively, south to north (Ratschbacher et al. 1989; Linzer et al. 1995; Neubauer et al. 2000). In the Eocene, the NCA were detached from their basement and thrust over the Rhenodanubian Flysch and Helvetic domain resulting in a wide thin-skinned tectonic nappe complex (Linzer et al. 1995; Neubauer et al. 2000).

The rheologically weak evaporitic succession at the base of the NCA—the Haselgebirge Formation—served as one of the major detachment levels. Halite, mudrock and subordinate anhydrite and polyhalite form an evaporitic mélange. This rock type is called “Haselgebirge”. In the salt mines, the average halite content ranges from ca. 30 to 65 vol% (Schauberger 1986; Leitner et al. 2011). Metre- to decimetre-sized isolated pieces of anhydrite and polyhalite rocks are often aligned parallel to the foliation.

These evaporites were deposited in a sabkha-like environment during the Late Permian and Lower Triassic (Klaus 1965; Spötl and Pak 1996; Spötl 1988b, c) within an aborted rift of the Tethys Ocean (Spötl 1989a). Rocksalt and mudrock were initially deposited in alternating layers of variable thickness.

Salt has been mined in central sectors of the NCA (Salzkammergut, Fig. 1) for more than 3,000 years (Stöllner 2003; Grabner et al. 2007). The mines and their surroundings are well described: Altaussee, Austria (Proisl 2003; Gawlick et al. 2007; Hofer and Klade 2010); Berchtesgaden, Germany (Pichler 1963; Kellerbauer 1996; Braun 1998); Bad Dürrnberg, Austria (Plöchinger 1990, 1996; Gawlick and Lein 2000); Hall in Tirol, Austria (Schmidegg 1951; Spötl 1988d, 1989b); Bad Ischl, Austria (Mayrhofer 1955; Medwenitsch 1957); Hallstatt, Austria (Schauberger 1931, 1949; Spötl 1987; Habermüller 2005; Gawlick and Schlagintweit 2006; Suzuki and Gawlick 2009).

## Materials and methods

Samples were collected in the underground mines, thin sections were prepared and analysed in the context of a broader polyhalite/anhydrite study, five thin sections are used in tables and pictures of this study.

### Deformation of halite cubes

**Fig. 2:**
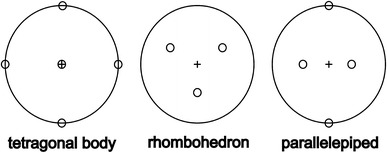
Possible deformation of a cube (Görgey 1912). *Empty circles* plot the normal of each surface plane of a cube in its relation to the normal of the compression plane (marked with a *cross*). Schmidt projection (*lower hemisphere*)

The plastic deformation of solid halite needs special attention with regard to the deformation of euhedral halite cubes. Görgey (1912) provided the first model of the possible deformation of cubes in general: if one of the fourfold axes is oriented perpendicularly to the compression plane, a tetragonal body develops (special case of a cuboid). If the compression acts along a threefold axis, the result is a rhombohedron. In case of deformation along one of the twofold axes, a parallelepiped develops (exposing parallelograms in cross sections; Fig. 2). Monocline and tricline symmetries can be deduced by a combination of two or three of these cases.

Intracrystalline effects on dislocation mobility or lattice diffusion contribute to the enhanced deformation of halite crystals. Impurities such as molecules of water enhance the start of dislocation glide, and the initiation of climb of dislocations to overcome obstacles. Effects of water on dislocation glide have been demonstrated for quartz (e.g. Post et al. 1996; Morgan and Law 2004), olivine (e.g. Jung and Karato 2001) and halite (Shlichta 1962; Pennock et al. 2006).

Halite already deforms at low pressure and low temperature by dislocation glide. For halite from Hengelo, the maximum overburden was ca. Five hundred metres and the estimated temperature did not exceed 50 °C. Based on the mechanism of dislocation glide, subgrains in halite developed, which allowed calculation of a differential palaeostress of 0.45–0.97 MPa (Schléder and Urai 2005). Previous investigations of natural rocksalt using the same piezometer revealed low differential palaeostresses at eight different salt deposits in America and Europe. Most of them were below 1 MPa (Carter et al. 1982).

Schléder et al. (2008) estimated low differential stresses of ca. 0.1–0.3 MPa for solution-precipitation creep of fine-grained halite of ca. 1 mm. The strong influence of mudrock or other non-halite materials on solution and precipitation of halite was observed in several experiments (Martin et al. 1999; Schutjens and Spiers 1999; Lohkämper et al. 2004). In a series of experiments, pressure solution was determined to start already at differential stresses of ca. 0.1 MPa (Hickman and Evans 1995).

### High-resolution X-ray computed tomography (HRXCT)

The aim of the method was to ascertain the palaeostress orientation relative to the sedimentary layers. HRXCT on mudrock with halite hopper crystals was carried out at the High-Resolution X-ray Computer Tomography (CT) Facility, University of Texas at Austin. A specimen from Altaussee (ALT-4D) with ca. 170 distinct halite hopper crystals and a recognisable sedimentary layering was examined. Scanning parameters were as follows: P250D, 450 kV, 1.5 mA, small spot, 1 brass filter, air wedge, 130 % offset, 64 ms integration time, slice thickness = 0.25 mm, S.O.D. 624 mm, 1800 views, 1 ray averaged per view, 1 sample per view and inter-slice spacing = 0.25 mm to produce 158 Tiff images (1024 × 1024 pixels, 16 bit per pixel) (for methodology, see Ketcham 2005). The Tiff images showed a perturbing noise. Standard classification digital methods were insufficient, so the halite hopper crystal contours were manually digitised in each slice. With the computer program Windicom (O‘Connor et al. 2009), the 158 images were stacked to reconstruct, by thresholding, a total of 64 deformed halite cubes in three dimensions. A best-fit ellipsoid was calculated and inscribed into each body. After inspection of reconstruction results, 52 deformed cubes were selected for calculation of the average strain ellipsoid. The axes of the ellipsoid related to the orientation of the pile of Tiff images. For re-orientation, the coordinates of the visible sedimentary layers of mudrock were traced on the Tiff images using the program ImageJ. From this data, two sedimentary layers were interpolated as surfaces by the program Surfer (Golden Software Inc. 2009). For quantifying the orientation of the main stress relative to the sedimentary layering, the average strain ellipsoid and the surfaces defining the sedimentary layers were re-combined in a single reference system in AutoCAD (AutoDesk).

### Electron microprobe analysis (EMPA)

EMPA on anhydrite was carried out on a JEOL electron microprobe (JXA-8600) at the Department Geography and Geology, University of Salzburg, using a wavelength dispersive system. Because sulphates are unstable under the electron beam, we used an acceleration voltage of 15 kV and a low sample current of 20 nA. The beam was defocused to a spot of 15 μm. Natural and synthetic mineral standards were used to calibrate the microprobe, and raw data was reduced using standard ZAF correction. Detection limits for Na_2_O, MgO, K_2_O, FeO, MnO and SrO are 0.02, 0.03, 0.03, 0.06, 0.07 and 0.03 wt%, respectively.

### ^40^Ar/^39^Ar dating

^40^Ar/^39^Ar techniques largely follow descriptions given in Handler et al. (2004) and Rieser et al. (2006). Preparation of the samples before and after irradiation, ^40^Ar/^39^Ar analyses, and age calculations were carried out at the ARGONAUT Laboratory of the Department of Geography and Geology at the University Salzburg. The samples were crushed carefully with a hammer. The material was washed with distilled water to dissolve halite, whose Cl-ions produce undesired Ar isotopes during irradiation, and dried with isopropanol. By comparison, the 200–250 μm fraction was separated under the microscope. Mineral concentrates were packed in aluminium-foil and placed in quartz vials. For calculation of the *J* values, flux-monitors were placed between each 4–5 unknown samples. The sealed quartz vials were irradiated in the MTA KFKI reactor (Budapest, Hungary) for 16 h. Correction factors for interfering isotopes were calculated from 45 analyses of two Ca-glass samples and 70 analyses of two pure K-glass samples and are as follows: ^36^Ar/^37^Ar_(Ca)_ = 0.000225, ^39^Ar/^37^Ar_(Ca)_ = 0.000614, ^38^Ar/^39^Ar_(Ca)_ = 0.011700 and ^40^Ar/^39^Ar_(K)_ = 0.0266. Variation in the flux of neutrons were monitored using the DRA1 sanidine standard for which an ^40^Ar/^39^Ar plateau age of 25.03 ± 0.05 Ma was originally reported (Wijbrans et al. 1995). Here, we use the revised value of 25.26 ± 0.05 Ma (Hinsbergen et al. 2008).

^40^Ar/^39^Ar analyses were carried out using a UHV Ar-extraction line equipped with a combined MERCHANTEK™ UV/IR laser system, and a VG-ISOTECH™ NG3600 mass spectrometer. Stepwise heating analyses of samples were performed using a defocused (~1.5 mm diameter) 25 W CO_2_-IR laser operating in Tem_00_ mode at wavelengths between 10.57 and 10.63 μm. The NG3600 is an 18 cm radius 60° extended geometry instrument, equipped with a bright Nier-type source operated at 4.5 kV. Measurements were performed on an axial electron multiplier in static mode. Peak-jumping and stability of the magnet was controlled by a Hall-probe. For each increment the intensities of ^36^Ar, ^37^Ar, ^38^Ar, ^39^Ar, and ^40^Ar were measured, the baseline readings on mass 34.5 were subtracted. Intensities of the peaks were back-extrapolated over 16 measured intensities to the time of gas admittance either by a straight line or a curved fit, depending on the intensity and type of the pattern of the evolving gas. Intensities were corrected for system blanks, background, post-irradiation decay of ^37^Ar and interfering isotopes. Isotopic ratios, ages and errors for individual steps were calculated following suggestions by McDougall and Harrison (1999) and Scaillet (2000) using decay factors reported by Steiger and Jäger (1977). Definition and calculation of plateau ages was carried out using ISOPLOT/EX (Ludwig 2003).

## Results

### Mudrock, halite hopper crystals and anhydrite cubes

**Fig. 3:**
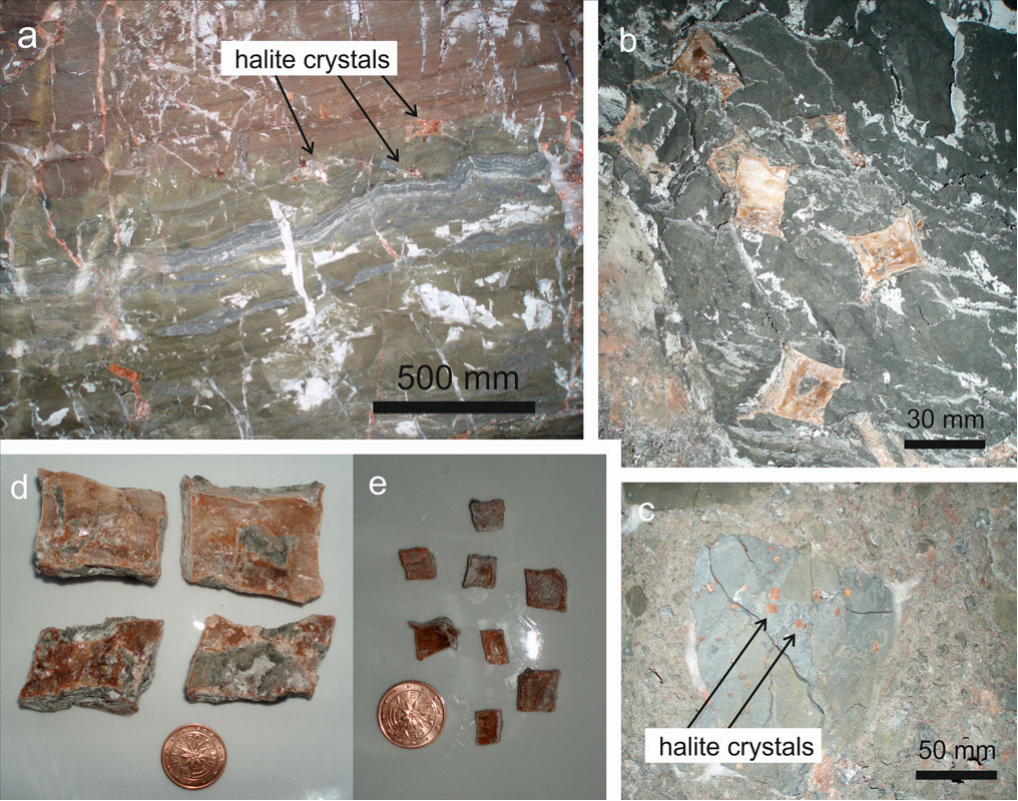
**a** *Green* and *red* mudrock with layers of gently folded dark anhydrite. Patches and veins consist of *white* and *red* halite. Halite cubes are marked by *arrows* (site BDG-28). **b**, **c** Halite cubes in mudrock. **d**, **e** Euhedral, deformed halite crystals

Halite hopper crystals were taken from blocks of mudrock. In representative samples, halite cubes range from 2 to 4 mm to 10–30 mm in size (Fig. 3). The biggest salt cubes in Berchtesgaden and in Altaussee were ca. 60 mm (edge length). The cubes usually occur within groups of similar size in blurred layers, whereby the single halite hopper crystals do not touch each other. The outer shapes of the crystals are cuboids, rhombohedrons or rhombic prisms. The halite hopper crystals show concave crystal surfaces with pronounced edges (hopper shape), whereby the surfaces are often stepped on a small scale. The larger the diameter of the “cubes”, the more elongated their edges. Cubes of more than 5 mm in diameter nearly always show elongated edges. A red coating surrounding the cubes causes the typical red colour, but internally the halite crystals are clear. Some of the hopper crystals, especially the bigger ones, enclose fragments of mudrock.

**Fig. 4:**
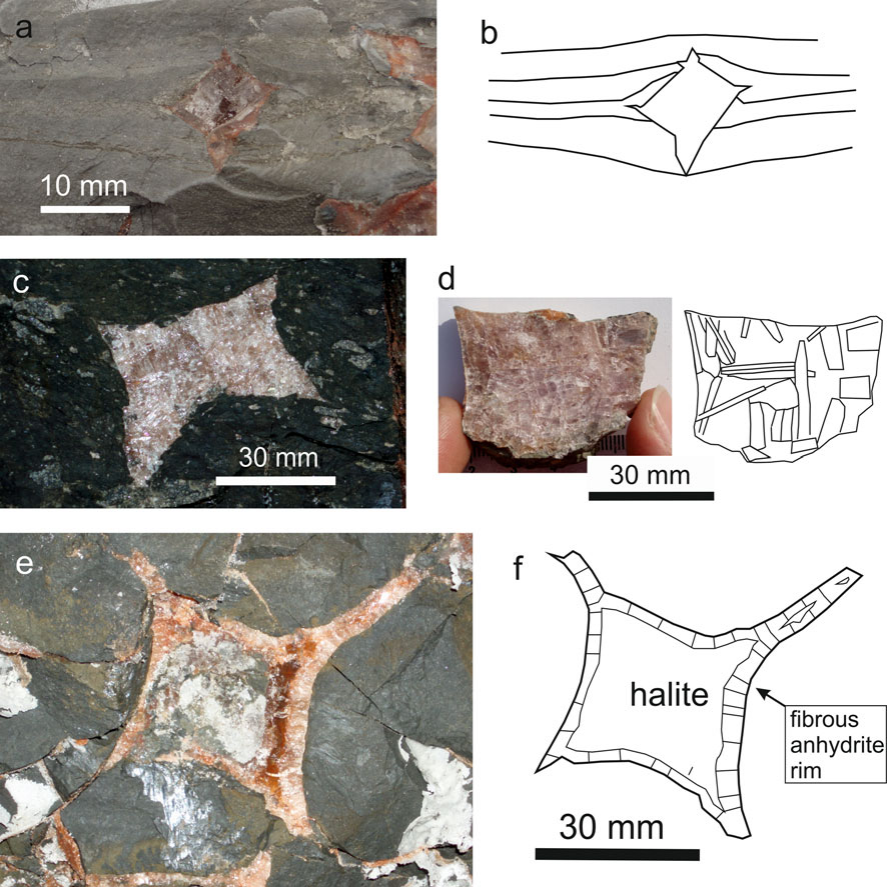
**a**, **b** Displacive halite cube. Note thickening of sediment layers towards the cube’s surface (Bad Dürrnberg). **c** Anhydrite cubes in undeformed mudrock of “bedded nodular” anhydrite structure (nomenclature after Maiklem 1969); site BDG-62. **d** Lath-shaped anhydrite crystals within a halite cube; site BDG-61. **e**, **f** Halite cube with strongly elongated edges surrounded by an anhydrite rim; site BDG-62

Most samples expose no sedimentary layering; however, samples with visible millimetre-thick layering were used to study the relation of halite cubes to the surrounding mudrock. The layers thicken towards the surface and end at the halite cube’s surface. This truncation and disruption of sedimentary features is typical for the displacement of mud matrix (Fig. 4a, b). Nine cubes expose both features, whereas two cubes only truncate the layering without disturbance.

Anhydrite cubes usually occur in fragments of internally undeformed mudrock. The diameter of the cubes ranges from 7 mm to 200 mm (typically 30–40 mm) and the cubes often show elongated edges (hopper shape). At sites BDG-61 and BDG-62, where most samples were collected, all cubes are cuboid, rhomboedric or prismatic. Their rose-coloured lath-shaped anhydrite crystals range from 3 mm to ≥20 mm in length showing no preferred orientation but a growth direction from the outer parts towards the centre (Fig. 4c, d). Mudrock inclusions are common, mostly between the crystals, but also in regions parallel to the rim of the cubes. Locally, polyhalite is present between anhydrite grains.

Some cubes are composed of halite and anhydrite. One particular sample from site BDG-62B is a platy cube with elongated edges, 30 mm in size. A clear halite crystal in the inner part is surrounded by fibrous anhydrite grains, which are orientated parallel to each other, and perpendicularly to the cube’s surface. A red coating in present between halite and anhydrite, which gives the internally clear halite cube its red colour (Fig. 4e, f).

### Polyhalite

**Fig. 5:**
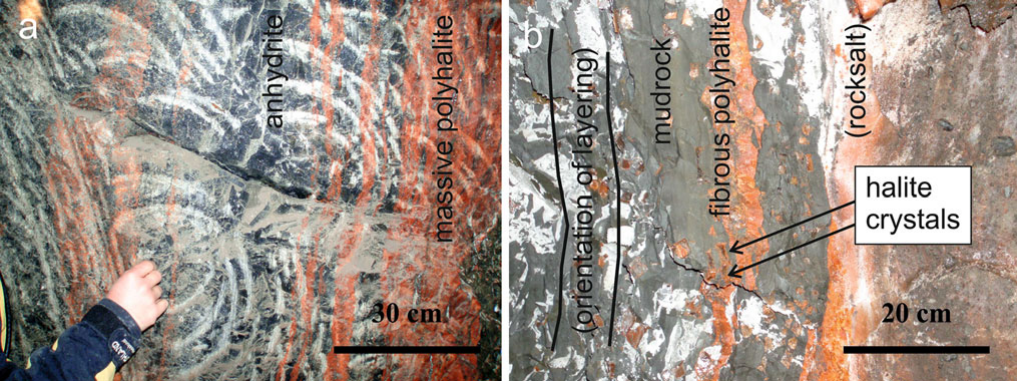
**a** Polyhalite forming reaction rims within dark-layered anhydrite. **b** Fibrous polyhalite overgrowing halite cubes. The polyhalite layer is parallel to the layering of the mudrock

Polyhalite in the Alpine salt rock is present in various rock types: (1) Polyhalite within rocksalt forms microscopic crystals and aggregates, which are evenly distributed within the rocksalt matrix. It gives the rocksalt its characteristic orange–red appearance; (2) When polyhalite is present within anhydrite rock, it occasionally forms reaction rims of parallel stripes (Fig. 5a). In the anhydrite matrix, polyhalite forms fibres or round crystals, the latter locally showing cores and rims. A gradual transition exists between polyhalite in anhydrite and polyhalite in mudrock. Polyhalite layers in mudrock range in thickness from millimetres to several decimetres and are parallel to the sedimentary layering of the mudrock. Anhydrite crystals are a typical feature of polyhalite within mudrock, which form laths of several centimetres in length partly aligned parallel to the sedimentary layers/foliation; (3) Veins with fibrous polyhalite exist within mudrock and are parallel to the sedimentary layers in most cases (Fig. 5b). The lowest stress orientation was thus vertical, a phenomenon, which was described in terms of hydraulic fracturing and/or crystallisation power for different minerals (e.g. Gustavson et al. 1994; Cosgrove 2001; Hilgers and Urai 2005; Philipp 2008; Rodrigues et al. 2009).

### Mineral growth succession of halite, anhydrite and polyhalite

**Fig. 6:**
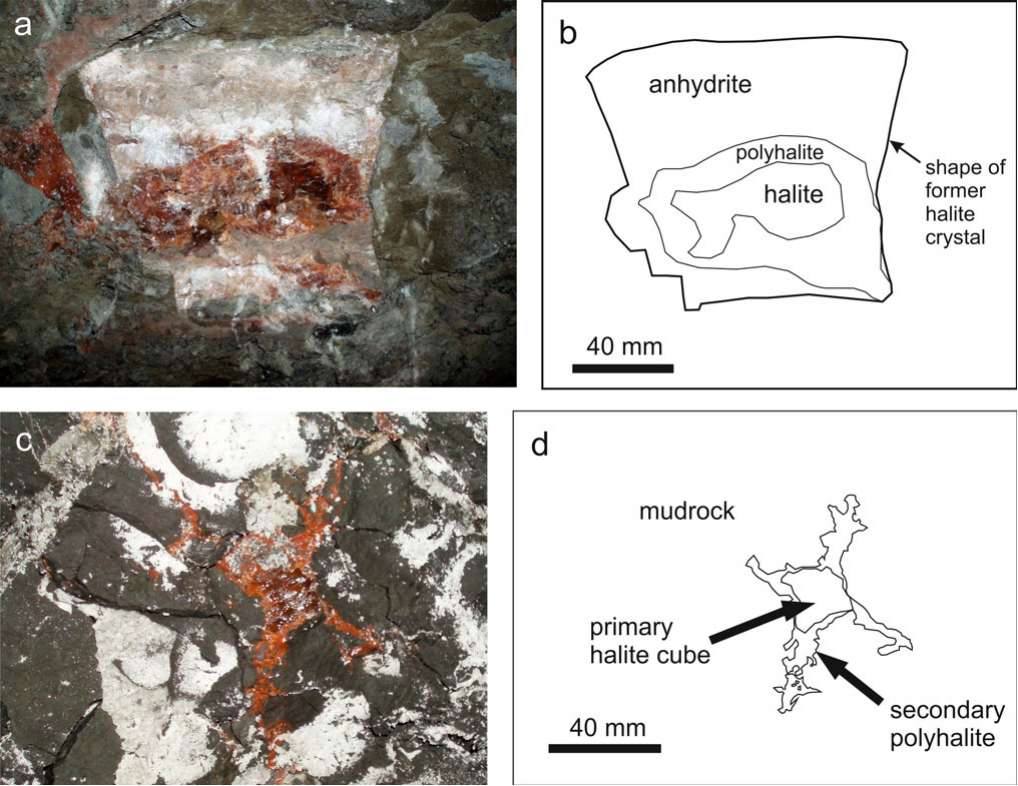
**a**, **b** Photo and sketch of anhydrite, polyhalite and halite; site BDG-12. **c**, **d** Photo and sketch. The elogated edges of halite are completely composed of polyhalite; site ALT-28

The large hopper-shaped cube of sample BDG-12A from Berchtesgaden, with an edge length of ca. 12 cm, contains, from the outside to the inside, anhydrite, polyhalite and halite (Fig. 6). In Altaussee, we found halite hopper-shaped cubes of up to 5 cm in edge length, exposed within nearly undeformed mudrock at the Franzberg level, site ALT-28. Other than in Berchtesgaden, the stage of anhydrite replacing halite is missing. However, at the margins, the halite is partly substituted by polyhalite.

**Fig. 7:**
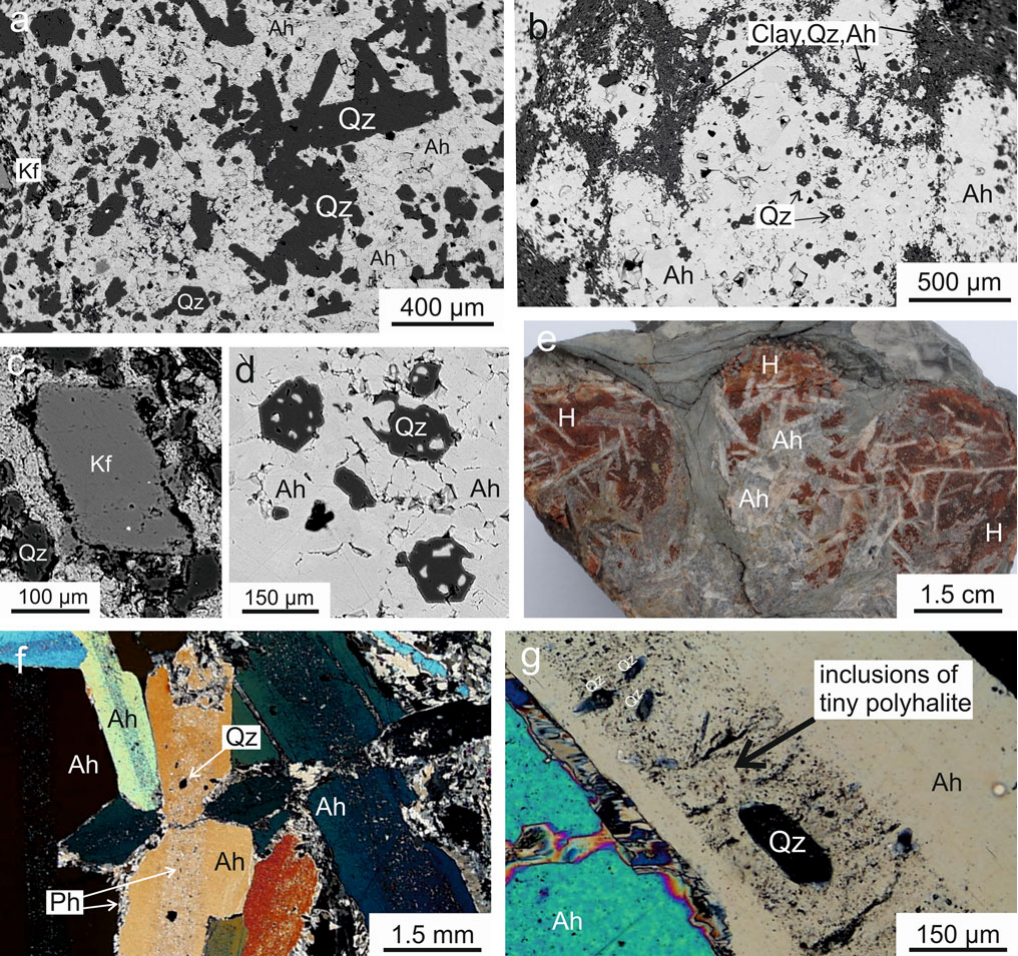
**a** Authigenic quartz and subordinate K-feldspar in anhydrite matrix. BSE image, sample BDG-28D. **b** Skeletal growth of euhedral quartz within anhydrite nodules. BSE image, ALT-53. **c** Detail of a, authigenic feldspar. **d** Detail of b. **e** Halite-anhydrite-polyhalite nodules in mudrock, site BDG-28. **f**, **g** Lath-shaped anhydrite comprises growth zones with tiny polyhalite and euhedral quartz. Thin section, crossed polarizers, sample BDG-28A. *Ah* anhydrite, *H* halite, *Kf* feldspar, *Qz* quartz, *Ph* polyhalite

A similar type of associated halite, anhydrite and polyhalite was found within halite-anhydrite-polyhalite nodules within mudrock. These nodules occurred in all three salt mines. At site BDG-28, the nodules are up to 10 cm in diameter and consist mainly of halite with euhedral lath-shaped anhydrite crystals. The large anhydrite grains may reach several centimetres in length. Under the microscope, they expose growth stages with zones of numerous tiny polyhalite grains (ca. 5–15 μm) and sometimes euhedral, authigenic quartz (50–150 μm). Polyhalite replaced the large anhydrite grains at the contact with halite, forming a seam around the large anhydrite grains. Authigenic quartz was also identified in the surrounding mudrock. There, they were overgrown by anhydrite. Euhedral K-feldspar grains are also present (Fig. 7).

### High-resolution X-ray computed tomography

**Fig. 8:**
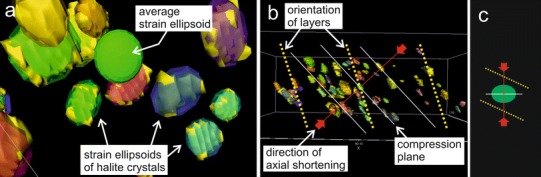
**a** Calculated strain ellipsoids and average strain ellipsoid. **b** Orientation of the average strain ellipsoid relative to the sedimentary layering within the sample and **c** reoriented in a gravity stress field

The axial ratios of the average strain ellipsoid are 1:1.7:1.9. The angle between the shortest axis of the strain ellipsoid (main compression orientation) and the surface normal to the sedimentary layering was determined to be 25 ± 5° (sample ALT-4D). The disk-shaped average strain ellipsoid is likely to result from compaction, that is, obliquely to the sedimentary layering (Fig. 8).

### Electron microprobe analysis

Anhydrite cubes revealed slightly elevated contents of Na of ca. 0.2 oxide wt%, whereas anhydrite rocks of nodular/chickenwire or of the dark-layered type from Berchtesgaden show Na values below 0.1 oxide wt%. The other elements show no significant differences (Table 1).

**Table 1:** Oxide wt% of anhydrite cube (BDG-62) compared to an anhydrite of nodular type (BDG-19) and to dark-layered anhydrite (BDG-28D)

Site	CaO	SO_3_	Na_2_O	MgO	K_2_O	FeO	MnO	SrO	Total
BDG-62	40.15	59.17	0.22	0.00	0.04	0.02	0.00	0.11	99.71
BDG-62	39.79	59.91	0.19	0.00	0.06	0.02	0.00	0.08	100.07
BDG-62	39.96	60.10	0.22	0.01	0.06	0.00	0.00	0.20	100.55
BDG-62	39.70	59.59	0.24	0.02	0.09	0.00	0.00	0.21	99.86
BDG-62	39.64	59.28	0.26	0.01	0.04	0.01	0.00	0.16	99.41
BDG-19	40.03	59.94	0.00	0.01	0.00	0.01	0.00	–	99.99
BDG-19	40.08	60.06	0.01	0.07	0.00	0.00	0.07	–	100.28
BDG-19	40.66	59.93	0.01	0.03	0.00	0.00	0.00	–	100.64
BDG-19	40.16	60.04	0.04	0.05	0.01	0.01	0.08	–	100.39
BDG-19	40.08	59.72	0.00	0.00	0.00	0.00	0.02	–	99.82
BDG-28D	39.65	58.97	0.07	0.16	0.01	0.03	0.00	–	98.87
BDG-28D	39.72	58.79	0.06	0.00	0.04	0.02	0.00	–	98.64
BDG-28D	39.52	60.08	0.04	0.05	0.03	0.00	0.00	–	99.72
BDG-28D	39.30	58.72	0.07	0.19	0.06	0.03	0.00	–	98.38
BDG-28D	39.06	59.22	0.04	0.00	0.03	0.04	0.01	–	98.40

### ^40^Ar/^39^Ar age dating of polyhalite

**Fig. 9:**
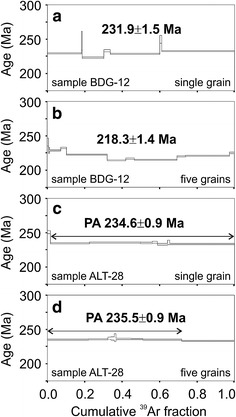
^40^Ar/^39^Ar release patterns of individual polyhalite crystals and polyhalite concentrates of samples BDG-12 and ALT-28

Total fusion and step heating ^40^Ar/^39^Ar dating has been performed on single grains and polygrains of the 200–250 μm fraction. Age spectra plots are shown in Fig. 9, an overview of analytical data is given in Table 2, and detailed data are compiled in Online Resource 1. Errors of ratios, *J* values and ages are reported on the 1−σ level (=standard deviation; 68.3 % confidence level). The argon release of polyhalite was limited to a narrow range of laser energy, and between 7 and 11 steps were measured. ^38^Ar/^39^Ar-ratios are the same as for K-monitors (=0.0117), proving that no significant Cl-concentrations were present in the samples. Therefore, no corrections for Cl-derived ^36^Ar were applied.

**Table 2:** ^40^Ar/^39^Ar analytical results

Polyhalite	^36^Ar/^39^Ar^a^	±	^37^Ar/^39^Ar^b^	±	^38^Ar/^39^Ar^a^	±	^40^Ar/^39^Ar^b^	±	^40^Ar^K^/^39^Ar*^c^	±	%^40^Ar*	%^39^Ar	Age (Ma)	± (Ma)
Sample: BDG-12_09s0069				200–250 μm		1 grain		*J* value: 0.007629 ± 0.000026						
1	0.000511	0.000088	0.515	0.011	0.0117	0.0002	17.910	0.029	17.77	0.04	99.3	18.2	229.4	0.9
2	0.003485	0.003668	0.406	0.251	0.0252	0.0047	20.310	0.394	19.29	1.15	95.1	0.4	247.7	13.8
3	0.000425	0.000095	0.493	0.020	0.0106	0.0003	17.351	0.035	17.24	0.04	99.5	11.5	222.9	0.9
4	0.000269	0.000365	0.474	0.105	0.0089	0.0007	18.150	0.046	18.08	0.12	99.7	3.5	233.1	1.6
5	0.000092	0.000089	0.499	0.014	0.0110	0.0002	17.736	0.018	17.72	0.03	100.0	26.5	228.8	0.8
6	0.001578	0.001504	0.488	0.250	−0.0027	−0.0027	19.919	0.129	19.46	0.46	97.8	1.0	249.8	5.6
7	0.000093	0.000037	0.515	0.007	0.0114	0.0001	18.052	0.025	18.04	0.03	100.0	38.9	232.6	0.8

Sample: BDG-12_09s0073				200–250 μm		5 grains		*J* value: 0.007629 ± 0.000026						
1	0.000736	0.001594	0.463	0.087	0.0141	0.0012	18.925	0.070	18.72	0.48	99.0	0.8	240.8	5.8
2	0.001638	0.001130	0.245	0.151	0.0146	0.0019	18.202	0.116	17.71	0.35	97.4	0.5	228.7	4.3
3	0.000259	0.000102	0.450	0.012	0.0110	0.0002	17.790	0.020	17.72	0.04	99.7	6.0	228.8	0.9
4	0.000326	0.000141	0.430	0.033	0.0101	0.0004	18.097	0.028	18.01	0.05	99.6	3.0	232.3	1.0
5	0.000770	0.000034	0.479	0.004	0.0114	0.0002	17.420	0.019	17.20	0.02	98.9	21.8	222.5	0.8
6	0.000231	0.000050	0.480	0.007	0.0112	0.0001	16.599	0.015	16.54	0.02	99.8	9.8	214.4	0.7
7	0.000575	0.000139	0.516	0.020	0.0112	0.0003	16.888	0.031	16.73	0.05	99.2	4.6	216.7	0.9
8	0.000155	0.000028	0.488	0.005	0.0112	0.0001	16.608	0.010	16.57	0.01	99.9	22.8	214.8	0.7
9	0.000745	0.000113	0.469	0.015	0.0117	0.0004	17.202	0.029	16.99	0.04	98.9	4.7	219.9	0.9
10	0.000236	0.000018	0.475	0.004	0.0113	0.0001	17.136	0.013	17.08	0.01	99.8	23.3	221.0	0.7
11	0.000353	0.000166	0.460	0.033	0.0100	0.0006	17.497	0.044	17.40	0.07	99.6	2.7	224.9	1.1

Sample: ALT-28_09s0098				200–250 μm		1 grain		*J* value: 0.007604 ± 0.000027						
1	0.001031	0.000492	0.484	0.072	0.0131	0.0011	19.910	0.100	19.62	0.18	98.6	1.7	250.8	2.3
2	0.000184	0.000042	0.499	0.012	0.0109	0.0001	18.301	0.041	18.26	0.04	99.9	20.9	234.5	0.9
3	0.000529	0.000050	0.490	0.008	0.0109	0.0001	18.488	0.020	18.34	0.02	99.3	27.2	235.6	0.8
4	0.000262	0.000210	0.455	0.039	0.0105	0.0003	18.368	0.026	18.30	0.07	99.7	6.3	235.0	1.1
5	0.001034	0.000290	0.543	0.025	0.0121	0.0005	18.686	0.059	18.40	0.10	98.6	2.7	236.2	1.5
6	0.001019	0.000110	0.510	0.021	0.0115	0.0003	18.438	0.081	18.15	0.09	98.5	5.5	233.2	1.3
7	0.001194	0.000876	0.640	0.086	0.0155	0.0014	18.709	0.077	18.38	0.27	98.3	1.1	236.0	3.3
8	0.000189	0.000022	0.490	0.006	0.0111	0.0001	18.211	0.019	18.17	0.02	99.9	34.7	233.4	0.8

Sample: ALT-28_09s0099				200–250 μm		5 grains		*J* value: 0.007604 ± 0.000026						
1	0.000490	0.000025	0.476	0.006	0.0115	0.0001	18.457	0.035	18.32	0.04	99.4	22.0	235.3	0.9
2	0.000305	0.000084	0.473	0.013	0.0116	0.0001	18.416	0.033	18.34	0.04	99.7	10.3	235.5	0.9
3	0.001209	0.000281	0.499	0.075	0.0099	0.0008	18.834	0.050	18.49	0.10	98.3	1.7	237.3	1.4
4	0.000223	0.000549	0.458	0.092	0.0116	0.0008	18.496	0.107	18.44	0.19	99.8	1.8	236.7	2.5
5	0.000479	0.001406	0.476	0.128	0.0125	0.0016	18.679	0.222	18.55	0.47	99.4	0.8	238.0	5.7
6	0.000121	0.000125	0.527	0.017	0.0102	0.0002	18.401	0.034	18.38	0.05	100.0	8.1	236.0	1.0
7	0.000230	0.000099	0.452	0.014	0.0107	0.0002	18.428	0.034	18.37	0.04	99.8	6.3	235.9	0.9
8	0.000179	0.000025	0.493	0.005	0.0113	0.0001	18.351	0.025	18.31	0.03	99.9	20.7	235.2	0.8
9	0.000316	0.000022	0.487	0.006	0.0116	0.0001	18.240	0.015	18.16	0.02	99.7	28.2	233.3	0.8

Errors of ratios, *J* values, and ages are at 1-sigma level

^a^Measured

^b^Corrected for post-irradiation decay of ^37^Ar

^c^Non-atmospheric ^40^Ar

Sample BDG-12A revealed an isochron age of 231.9 ± 1.5 Ma for the single-grain measurement, whereas the multi-grain measurement showed a slightly disturbed argon release pattern with an isochron age of 218.3 ± 1.4 Ma. We suggest that the single-grain result is geologically significant and that multi-grain age results either from a mixture of grains of different ages or some Ar loss after ca. 232 Ma.

The single-grain measurement of sample ALT-28 yielded a plateau age of 234.6 ± 0.9 Ma (98.3 % ^39^Ar released). The multi-grain measurement revealed a plateau age of 235.5 ± 0.9 Ma. The results from Altaussee are thus similar to those of the Berchtesgaden polyhalite.

## Discussion

### Halite hopper crystal formation and mud compaction

Gornitz and Schreiber (1981) observed isolated, euhedral halite cubes in soft mud in the Dead Sea, which had fallen dry. They attributed these hopper-shaped crystals to growth by displacement of mudrock just beneath the sediment surface.

In Alpine mudrock, visible sedimentary layers within the fine-grained matrix thicken towards the halite hopper crystal surface and are truncated (Fig. 4a, b). This feature can be explained by the displacement of soft mudrock (Gornitz and Schreiber 1981; Benison and Goldstein 2000). Mechanical compaction structures such as fractured fossils, deformation of the sediment layers around rigid objects, as well as chemical compaction structures such as stylolithes, or pressure solution at clast contacts (e.g. Füchtbauer 1988; Tucker 1991; Potter et al. 2005) are uncommon. However, mineral transformation and growth of anhydrite, authigenic quartz and polyhalite, indicate dissolution and re-precipitation.

The shear strength of consolidated mudrock transmitted the stress required to deform halite. The glide and climb mechanism of halite operates at less than 1 MPa differential stress at low temperatures (e.g. Schléder and Urai 2005). The presence of water accelerates the intracrystalline deformation (e.g. Roedder 1984). Compaction of the mud and deformation of the halite crystals was accompanied by the expulsion of excess pore water from the mud (e.g. Tucker 1991) and possibly, to a lesser degree, due to the transformation of gypsum to anhydrite (e.g. Langbein et al. 1982). However, the contribution of solution-precipitation cannot be definitely ruled out. The halite hopper crystals were most probably deformed in a simple overburden-dominated stress field. The resulting shape depends on the initial random orientation of the halite cubes, and cuboids, rhombohedrons and prisms develop (cf. Görgey 1912). Our X-ray CT observations on sample ALT-4D show a major finite strain orientation and two equal minimum and intermediate finite strain orientations. This axial shortening points to a compaction, where the main strain orientation was parallel to gravity. However, in sample ALT-4D, the main stress orientation is oblique to the sedimentary layering (Fig. 8).

Finally, rock stiffening by mechanical and chemical compaction (e.g. Thyberg and Jahren 2011) preserved the halite cubes from further compression and destruction.

### Anhydrite pseudomorphs after halite

Anhydrite cubes are interpreted as pseudomorphs after halite. Transformation clearly occurred after the halite cubes had reached their final size and shape (Fig. 4c–f). Calcium sulphate-rich solutions migrated into the cubes and replaced former halite. Arguments for this interpretation are as follows: (1) anhydrite crystals do not form cubes; (2) anhydrite crystals with elongated edges like halite are unknown; (3) transition phases of halite cubes partly replaced by fibrous anhydrite growing inward towards the centre were observed; and (4) electron microprobe analysis revealed slightly elevated concentrations of Na in anhydrite cubes hinting at a halite precursor.

The pore water, which dissolved halite, was obviously oversatured with respect to anhydrite. This dilution effect could reflect (i) dewatering of mud (rock), (ii) gypsum-to-anhydrite and/or carnallite-to-sylvite transformation or (iii) input of less concentrated brines from an external source. The latter option seems unlikely, since such water would have quickly become saturated with respect to halite given the abundance of halite in these rocks. The gypsum-to-anhydrite conversion would result in a release of 40 vol% structural water (Borchert and Muir 1964). The carnallite-to-sylvite transition releases 40 vol% of water (Borchert and Muir 1964). The primary amount of gypsum and carnallite is uncertain. Probably, the dehydration of these minerals contributed water, but would again become rapidly saturated with respect to halite. Therefore, scenario (i) seems the most reasonable one. Brines undersaturated with regard to halite migrated through the rock when mudrocks and anhydrite rocks were intact and not yet totally destroyed by the formation of the Haselgebirge mudrock-halite tectonite.

### Polyhalite pseudomorphs after halite

Polyhalite was found in rocksalt, anhydrite and mudrock. When polyhalite crystallised, brines containing K^+^, Mg^2+^ and Ca^2+^ migrated through the rock. The solution was also saturated with respect to halite since substitution of halite by polyhalite is uncommon. In Hallstatt, the relatively high Rb content—0.0012 instead of 0.0001 wt%—suggests that Rb-rich K-salt minerals dissolved and polyhalite precipitated from these brines (Kühn 1972). Large anhydrite crystals (several centimetres) from common polyhalite rock, and large anhydrite crystals from halite-anhydrite-polyhalite nodules, contain numerous solid inclusions of polyhalite. The phantom-like growth stages suggest an initial rapid growth of the large anhydrite crystals simultaneously with the tiny polyhalite inclusions (Fig. 7f, g). The crystallisation of large anhydrite crystals and polyhalite should thus be seen in a general mobilisation of fluids. We suppose that polyhalite formed from subsequent enhanced fluid migration. Mudrock provided water by ongoing dewatering, while potassium and magnesium were dissolved from primary salt minerals. Mudrock had been sealing the evaporation cycle and repeated ingress of sea water did not result to the accumulation of potassium-rich salt levels. When these migrating fluids interacted with sulphates, polyhalite precipitated. Our ^40^Ar/^39^Ar analyses date the polyhalite from within the retaining shapes of deformed halite hopper-shaped cubes from two localities to ca. 235–232 Ma (Ladinian; Ogg et al. 2010). This is 20–25 Ma younger than the age of evaporite deposition (uppermost Permian; e.g. Klaus 1965, Spötl and Pak 1996).

However, what were the pressure/temperature conditions in this environment? The presence of water—polyhalite contains crystal water—and low pressures may have contributed to the development of polyhalite. The growth of polyhalite in layers parallel to the sedimentary bedding indicates that the overburden was more or less tectonically undestroyed. For the mobile salt bodies, buoyant salt rise starts at ca. 1,600–3,000 m overburden (Hudec and Jackson 2007). At 230 Ma, the overburden reached ca. 800 m at most (e.g. Rantitsch and Russegger 2005), but could have been locally only some hundred metres. The lowest stress orientation and growth direction of fibrous polyhalite in veins was vertical, a phenomenon, which was described in terms of force of crystallisation for different minerals (Hilgers and Urai 2005).

With regard to the temperature during anhydrite/polyhalite crystallisation, an interesting fact is the coeval crystallisation of authigenic quartz. In some samples, authigenic quartz is included within the large anhydrite crystals, which is also present in the surrounding mudrock (Fig. 7). Authigenic quartz was described in clastic strata related to Zechstein and Haselgebirge salt deposits (Grimm 1962; Franz 1967; Nachsel 1966, 1969; Spötl 1988a). Based on experiments at the beginning of the twentieth century, Grimm (1962) postulates the precipitation of SiO_2_ from fluids under saline conditions and a post-sedimentary, diagenetic growth. However, the chemical reaction of smectite + K^+^ = illite + silica + H_2_O might also be responsible for the formation of authigenic quartz (Peltonen et al. 2009; Thyberg et al. 2010; Thyberg and Jahren 2011). This well-known smectite-to-illite (S–I) transition of mixed-layer clay minerals (S–I-transition) happens between 60 and 90 °C (e.g. Deng et al. 1996; Couzens-Schultz and Wiltschko 2000; Charpentier et al. 2003; Saffer et al. 2008). Thus, temperatures of 60–90 °C could have been reached in Alpine mudrock in the rocksalt deposits of Berchtesgaden and Altaussee. A fluid inclusion study failed in terms of the smallness of the fluid inclusions within authigenic quartz (pers. comm. S. Borojević-Šoštarić, University of Zagreb).

Another plausible reason to assume elevated temperatures during the formation of polyhalite is that halite is a very good heat conductor. High palaeo-temperatures of ca. 380 °C have been measured in bitumen in the Ara Salt, Oman (Schoenherr et al. 2007). In the Lesser Himalaya, India, temperatures of up to ca. 300 °C were measured in fluid inclusions in anhydrite crystals (Singh and Singh 2010). During the Upper Jurassic (?) to Cretaceous, Alpine orogeny temperatures of ≥200 °C were reached (e.g. Kralik et al. 1987; Wiesheu and Grundmann 1994; Spötl and Hasenhüttl 1998; Spötl et al. 1998). Relatively high geothermal gradients were also reported from salt bodies in regions where no orogeny took place. In Germany, at depths of 500–800 m, a palaeo-temperature of 70–80 °C was estimated for Neuhof (Schléder et al. 2008), and 60 °C were reported from the abandoned underground salt mine in Wittelsheim in the southern Rhine graben and the active mine Sigmundshall near Hannover (pers. comm. K + S AG).

The presence of mudrock, anhydrite and possibly early elevated temperatures is a reasonable explanation for the appearance of polyhalite. A thermal heating would chronologically fit well with the further opening and rifting of Pangea (e.g. Blakey 2008). According to Kozur (1991) and Stampfli and Borel (2002), the Neotethys opened as far as the present eastern Mediterranean Sea. Between 240 and 220 Ma the subduction of the northern Palaeotethys triggered the opening of backarc oceans from Austria to China, including the Meliata, Maliac and Pindos Oceans.

## Conclusions

- Halite hopper crystals preserved in mudrocks of the Alpine Haselgebirge Formation deformed by mud(rock) compaction and the concomitant release of water. Thereby, cubes transformed into rhombohedrons, cuboids and prisms.
- Anhydrite cubes are pseudomorphs after halite implying a dilution of the pore water probably supplied by ongoing mudrock compaction and subordinately by the gypsum-to-anhydrite conversion.
- ^40^Ar/^39^Ar data show that polyhalite crystallised at ca. 235–232 Ma.
- The presence of mudrock, with its dewatering and also sealing capacity, the presence of anhydrite and possibly a thermal heating event provide a plausible explanation for the appearance of polyhalite and the absence of other K-bearing evaporite minerals.
- As indicated from intact mudrock bedding, polyhalite crystallised in shallow levels similar to vertical fibrous polyhalite crystals in bedding-parallel veins.
- The crystallisation of polyhalite in deformed halite-anhydrite cubes took place during rift-to-drift transition during opening of the Meliata oceanic basin.

## Electronic supplementary material

Below is the link to the electronic supplementary material.

Table 1 Detailed ^40^Ar/^39^Ar step heating data for polyhalite (XLSX 1012 kb)
